# The interface between cholinergic pathways and the immune system and its relevance to arthritis

**DOI:** 10.1186/s13075-015-0597-2

**Published:** 2015-03-31

**Authors:** Robin M McAllen, Andrew D Cook, Hsu Wei Khiew, Davide Martelli, John A Hamilton

**Affiliations:** Florey Institute of Neuroscience and Mental Health, University of Melbourne, Parkville, Victoria 3010 Australia; Department of Medicine, Royal Melbourne Hospital, University of Melbourne, Parkville, Victoria 3010 Australia

## Abstract

The nervous and immune systems are likely to be interacting in arthritis, with the possible involvement of both neural and non-neural cholinergic transmission. Centrally acting muscarinic agonists, electrical stimulation of the vagus and treatment with nicotinic receptor agonists can all act systemically to reduce inflammation, although the responsible pathways are incompletely understood. While this ‘cholinergic anti-inflammatory pathway’ is widely viewed as a significant pathophysiological mechanism controlling inflammation, the evidence supporting this view is critically reviewed and considered inconclusive; an alternative pathway via sympathetic nerves is implicated. This review also discusses how cholinergic pathways, both neural and non-neural, may impact on inflammation and specifically arthritis. Nicotinic agonists have been reported to reduce the incidence and severity of murine arthritis, albeit an observation we could not confirm, and clinical studies in rheumatoid arthritis have been proposed and/or are underway. While the therapeutic potential of nicotinic agonists and vagal stimulation is clear, we suggest that the ‘cholinergic anti-inflammatory pathway’ should not be uncritically embraced as a significant factor in the pathogenesis of rheumatoid arthritis.

## Introduction

**Figure 1:**
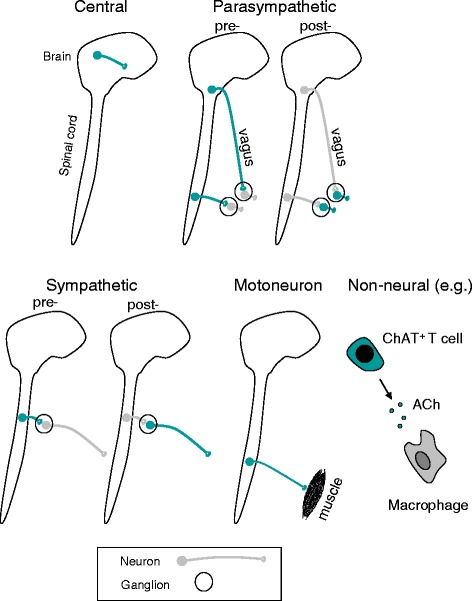
**Schematic summary of the types of cholinergic pathway.** The following cholinergic pathways are highlighted in green in successive diagrams: (i) central nervous, (ii) preganglionic and postganglionic parasympathetic (cranial and sacral), (iii) preganglionic and postganglionic sympathetic, (iv) somatic motoneuron and (v) non-neural (showing an example of a cholinergic cell-cell interaction). All somatic motoneurons, all sympathetic and parasympathetic preganglionic neurons and most parasympathetic postganglionic neurons are cholinergic; the remainder are subsets. ChAT^+^ = choline acetyl transferase-positive; that is, acetylcholine (ACh) expressing.

Nervous and immune system interactions are likely to be occurring in arthritis, as exemplified by the observation that hemiplegic patients do not develop psoriatic arthritis on their denervated side [1]. In this review we will try to identify relevant cholinergic pathways in the physiological and pathophysiological sense - those that are actually used by the body *in vivo*. Recently it has become increasingly apparent that, as well as responding to transmitters released by autonomic nerves, cells of the immune system may express and intercommunicate by these same transmitters [2,3]. The different types of cholinergic pathway are shown in Figure 1. These are (i) central nervous, (ii) preganglionic and postganglionic parasympathetic, (iii) preganglionic and postganglionic sympathetic, (iv) somatic motoneuron and (v) non-neural (cell-cell interaction). All somatic motoneurons, all preganglionic neurons and most postganglionic parasympathetic neurons are cholinergic. Only a minority of central and sympathetic postganglionic neurons are cholinergic. The latter supply targets such as the sweat glands and are unlikely to be directly involved in modulating immune function [4]. Vagal afferent neurons are not included since they are generally not cholinergic (see below).

The cholinergic neurons that influence immune function may do so directly by the actions of synaptically released acetylcholine on immune cells, or indirectly by synaptically exciting other neurons with the same or different transmitters (for example, noradrenaline). They do not directly innervate joints. The immune mechanisms to be considered will focus on the control of inflammation and, where specific information exists, its relevance to arthritis. We will then review the pharmacological actions of cholinergic agonists on monocytes/macrophages and report new findings on how they affect murine collagen-induced arthritis.

## Central neural cholinergic pathways

Around the turn of the century Borovikova and colleagues, working in the Tracey laboratory, showed that an anti-inflammatory drug, CNI-1493, was effective in suppressing both local inflammation (carrageenan-induced paw oedema) and systemic inflammation (hypotension and the inflammatory cytokine response to intravenous treatment with lipopolysaccharide (LPS)) in anaesthetised rodents [5,6] - the strength of a systemic inflammatory response to a stimulus such as LPS is often measured by the levels of the pro-inflammatory cytokine, tumour necrosis factor (TNF) [7]. These workers found that CNI-1493 was many times more potent in suppressing inflammation when given into the cerebral ventricles than when given intravenously, showing that its site of action was in the central nervous system [5]. Later, it was found to act via central muscarinic receptors, and that other muscarinic agonists with central neural actions had similar anti-inflammatory effects [8]. Furthermore, treatment with the centrally acting cholinesterase inhibitor, galantamine, was found to suppress acute systemic inflammation [9]. This last finding suggests that central cholinergic neurons (Figure 1) tonically release acetylcholine close to the muscarinic receptors that drive the anti-inflammatory action.

The output pathway stimulated by these central muscarinic actions was found to run in the vagus nerves (discussed below). The anti-inflammatory action of these muscarinic agonists was blocked by centrally acting muscarinic antagonists but not by atropine methyl nitrate, which does not cross the blood–brain barrier [8]. These observations are in line with those of others who concluded that central but not peripheral muscarinic receptors have an anti-inflammatory action [10].

Muscarinic receptors in the spinal cord have also been shown to exert anti-inflammatory actions. Yoon and colleagues [11] found that intrathecal injection of a muscarinic M2 receptor agonist suppressed peripheral inflammation in the zymosan-treated air pouch model in mice. In this case, however, the anti-inflammatory action was mediated by sympathetic nerves to the adrenal medulla and the release of adrenal catecholamines [12].

## The ‘cholinergic anti-inflammatory pathway’ and the vagus

Following their demonstration that the anti-inflammatory actions of CNI-1493 were mediated by the vagus nerves, Tracey’s group and subsequently others have shown that electrical stimulation of the peripheral end of either the left or the right cut vagus has strong systemic anti-inflammatory actions [3,5,13,14]. In most cases the inflammatory cytokine response to systemic LPS treatment has been measured in anaesthetised rodents, and this is reduced substantially by vagal stimulation. These and other relevant actions of vagal stimulation are well reviewed elsewhere [15].

Surprisingly for an action mediated presumptively by postganglionic parasympathetic nerves (Figure 1), the anti-inflammatory effects of vagal stimulation are not blocked by muscarinic antagonists such as atropine methyl nitrate [8], although they are blocked by antagonists of β_2_ adrenoreceptors and are absent in mice lacking those receptors [16]. Nicotinic acetylcholine receptors (nAChRs) containing the α7 subunit (α7nAChR) were found to be essential for its action, because the effect was absent in mice lacking this subunit [17]. However, the site of those essential nicotinic receptors is uncertain (discussed in more detail in [18]). α7nAChR is predominantly expressed in neuronal tissues but also in several non-neuronal cell types such as immune cells (for example, monocytes, macrophages, lymphocytes), epithelial cells and adipocytes [19] (Figure 1). This vagally mediated anti-inflammatory action has been termed the ‘cholinergic anti-inflammatory pathway’ [7,15].

As discussed elsewhere [18], the pathway from the vagus to its anti-inflammatory action is complex and incompletely understood. It involves the spleen [20] and the splenic nerves [14], but the proposal [15] that it is mediated by a direct synaptic connection from the vagus to the splenic nerves (which are sympathetic) has been refuted [21]. A non-neural link (Figure 1), possibly mediated by acetylcholine-secreting T lymphocytes [3], seems to form an essential part of the pathway [18]. B lymphocytes can also produce acetylcholine, which is reported to control local neutrophil recruitment to the peritoneum in response to endotoxin [22]. Further, stimulation of the vagus has been found also to inhibit leukocyte migration in another site that it does not innervate - the carrageenan air pouch model of inflammation [23].

## The inflammatory reflex

The concept of an inflammatory reflex, in which the central nervous system responds to inflammatory stimuli and acts to limit peripheral or systemic inflammation, was clearly formulated by Tracey [7]. In parallel with this neural reflex, but slower to act, is the release of glucocorticoids by the hypothalamic-pituitary-adrenal axis [24,25]. These two mechanisms are seen as early regulators by which the body acts to moderate the strength of the inflammatory response to infection or injury.

It has been stated in a number of articles that the ‘cholinergic anti-inflammatory pathway’ constitutes the efferent neural arm of the inflammatory reflex [7,15]. The critical test for this idea is that when the proposed inhibitory pathway is disconnected (that is, by cutting the vagi to interrupt parasympathetic preganglionic transmission; Figure 1) this should exacerbate the strength of the inflammatory response to immune challenge. In the original paper describing the vagal pathway, this appeared to happen: Borovikova and colleagues [6] found that cutting the vagi in anaesthetised rats caused a 40% increase in their inflammatory response (measured by plasma levels of TNF) to a high dose of intravenous LPS. However, later studies from the same and other laboratories have failed to replicate this finding [26-29]. The possible reasons are discussed elsewhere [18]. We recently reinvestigated this question using the same paradigm - measuring the plasma TNF response to intravenous LPS in anaesthetised rats - and found that cutting the vagi had no effect on this measure of inflammation [28]. By contrast, sectioning of the splanchnic sympathetic nerves increased the TNF response to LPS five-fold. We concluded that the efferent arm of the inflammatory reflex runs not in the vagi but in the splanchnic sympathetic nerves [28]. This is in line with a substantial body of literature that implicates sympathetic nerves in the control of systemic inflammation [30-32]. Critically, however, our findings indicate that the ‘cholinergic anti-inflammatory pathway’ in the vagus is not activated endogenously by systemic inflammation, only by exogenous pharmacological or electrical means. This has implications for its potential role in inflammatory conditions such as arthritis (see below).

## Where vagotomy does affect inflammation

In contrast to the case with systemic inflammation, there is good evidence for the involvement of the vagus in modulating inflammation of the gut and abdominal viscera. For example, prior sectioning of the left cervical vagus has been shown to enhance the blood levels of inflammatory cytokines measured in mice 6 hours after the induction of septic peritonitis [33]. Unilateral vagotomy also worsens the severity of cerulean-induced pancreatitis and raises the levels of the associated circulating pro-inflammatory cytokines over a time course of days [34]. These and related findings on the vagal modulation of intestinal inflammation are ably reviewed elsewhere [35].

In keeping with the idea that there is some abdominal ‘local sign’ in the vagal anti-inflammatory influence, mild inflammation was detected in the lungs of the same mice that were given pancreatitis, yet the lung inflammation was unaffected by vagotomy [34]. Furthermore, in experiments where the lungs of rats were directly inflamed by exposure to diesel soot, vagotomy was actually found to reduce the lung inflammatory response, suggesting a vagal pro-inflammatory action in this tissue [36]. This pro-inflammatory action was blocked by atropine [36].

What remains unclear from these findings, however, is whether the protective actions of the vagus are mediated by parasympathetic efferent fibres (as in Figure 1) - that is, the ‘cholinergic anti-inflammatory pathway’ - or by vagal afferent fibres (or perhaps both). Some 80 to 90% of the nerve fibres that run in each vagus are not parasympathetic but are visceral afferents [37]. Critically, these are generally not cholinergic. They can have anti-inflammatory actions, as shown by the following examples. In animals given colitis (usually induced experimentally by trinitrobenzenesulfonic acid), vagotomy worsens the severity of the disease [38]. Selective destruction of vagal afferents with capsaicin treatment (which blocks traffic in a subset of afferent fibres while sparing autonomic efferents [39]) also worsens disease severity [40] and increases mortality [41], suggesting that vagal afferents normally have an anti-inflammatory action. In rats subjected to haemorrhagic shock or acute haemolysis, Luyer and colleagues [42,43] have demonstrated a dramatic protective effect of a high-fat diet. In haemorrhagic shock, the levels of pro-inflammatory cytokines, such as TNF and interleukin-6, were dramatically lower in the fat-fed animals and their intestinal barrier integrity was preserved [43]. This protection disappeared if the vagi were cut or the animals were given antagonists to cholecystokinin [42], which is released by lipid in the intestine and stimulates vagal afferents [44]. A high-fat diet was also found to reduce the damaging effect of haemolysed blood on kidney, liver and intestinal function. This protection also depended on the vagi and cholecystokinin receptors [45], indicating that a reflex mediated by vagal afferent fibres was responsible. In all these cases the protective effects of vagal afferents were blocked by systemic administration of nicotinic antagonists such as chlorisondamine or hexamethonium. These data indicate that the efferent pathway of the protective reflex triggered by vagal afferents is probably autonomic, but do not distinguish whether it is sympathetic or parasympathetic.

In summary, in contrast to the case in acute systemic inflammation, the vagi mediate an inhibitory action on abdominal inflammation. It is unclear, however, whether any of this protective action is mediated by vagal efferent fibres of the ‘cholinergic anti-inflammatory pathway’. There is strong evidence that vagal afferent fibres are involved, but there is no evidence yet proving that the reflex motor pathway is vagal rather than sympathetic. Indeed in the case of another reflex response to abdominal inflammation - gastroparesis following intestinal manipulation - it has been shown that the afferent pathway is vagal but the efferent pathway is sympathetic [46].

## The ‘cholinergic anti-inflammatory pathway’ and arthritis

The vagus nerve does not directly innervate the joints, so any action that it may have on arthritis must be indirect. Nevertheless, could a loss of control by the ‘cholinergic anti-inflammatory pathway’ play a role in maintaining arthritis? This hypothesis [47] was investigated in mice by van Maanen and colleagues [48], who found that unilateral cervical vagotomy caused only a non-significant trend to worsen the disease. Wu and co-workers [49] recently confirmed that unilateral vagotomy had no significant effect. On the other hand, collagen-induced arthritis was found to be exacerbated in mice lacking α7nAChR [50], suggesting that nicotinic receptors independent of the vagus could be relevant (discussed below). In humans, a large case–control study on data from nearly 200,000 patients on the Swedish inpatient register found that surgical vagotomy caused no excess risk of developing rheumatoid arthritis (RA) [51], although it did not investigate whether vagotomy affected disease severity.

On the other hand, several correlation studies have investigated a link between parasympathetic nerve activity to the heart (cardiac vagal tone) and inferred activity in the ‘cholinergic anti-inflammatory pathway’. High frequency heart rate variability (HF-HRV) and beat-to-beat variability of cardiac interval both measure respiratory sinus arrhythmia, which is an index of cardiac vagal tone. It is commonly used in measures of ‘sympathovagal balance’, a major determinant of health. Reduced heart rate variability has been described in RA and systemic lupus erythematosus patients [52]. In addition, heart rate variability correlated with RA disease severity [47] and was suppressed compared with that in normal controls. This is in line with evidence that HF-HRV is reduced in other inflammatory conditions and correlates inversely with inflammatory markers such as C reactive protein on a population basis [53].

To suggest that cardiac vagal tone reflects tone in the vagal fibres of the ‘cholinergic anti-inflammatory pathway’ [47] is a bold hypothesis, given that most evidence indicates that parasympathetic tone is organ-specific [4]. Bradycardia and penile erection, for example, are both parasympathetic nerve actions: trained athletes have strong cardiac parasympathetic tone and a slow resting heart rate but they do not generally walk around with a permanent erection! In the case of acute systemic inflammation, our evidence does not support the hypothesis. Cutting the vagi in LPS-treated rats revealed significant cardiac vagal tone (heart rate increased by 50 beats/minute) but no functional tone in the ‘cholinergic anti-inflammatory pathway’ (inflammation was not exacerbated) [28]. Whether cardiac vagal tone turns out to be a surrogate measure of tone in the ‘cholinergic anti-inflammatory pathway’ over the longer term remains to be proven. On the other hand it is well established that cardiac vagal tone (measured by HF-HRV) is directly suppressed by peripheral inflammatory stimuli [54], which act via the brain to alter autonomic function [55]. The raised pulse rate that accompanies fever is familiar to us all. Therefore, reduced cardiac vagal tone (HF-HRV) is a predictable consequence of peripheral inflammatory processes, and this would provide the most parsimonious explanation for why it varies inversely with inflammatory markers. Any causative relation remains unproven.

## Vagal stimulation in arthritis

Even if the endogenous role of vagal transmission in the aetiology of arthritis is uncertain, could there be a therapeutic role for vagal stimulation to relieve arthritis? It is known that vagal stimulation can suppress limb inflammation (carrageenan-evoked paw oedema) in the acute setting [5]. In the chronic setting, Zhang and co-workers [56] reported that a novel technique, ‘vagus nerve suspension’, resulted in a modest but significant amelioration of collagen-induced arthritis in rats over 2 to 5 weeks. The authors suggested that the technique caused chronic vagal stimulation, perhaps by a combination of mechanical irritation and local inflammation [56]. Without further investigation, however, it is unclear to what extent the effect of suspension on the vagus might be damage rather than stimulation, or what its mode of action on arthritis might be. Very recently, however, Levine and colleagues [57] showed convincingly that electrical stimulation of the left cervical vagus with chronically implanted cuff electrodes substantially reduced ankle swelling and histological measures of arthritis in rats with collagen-induced arthritis. The therapeutic effect was well developed within a week of stimulation treatment which, strikingly, was given for only 60 seconds per day [57]. Necessarily, both afferent and efferent vagal nerve fibres remained intact. Action potentials conduct in both directions so, besides confirming the finding, it is important for future studies to determine whether the therapeutic benefit is due to stimulating parasympathetic efferent fibres, visceral afferent fibres or both.

## Sympathetic preganglionic neurons in inflammation and arthritis

The evidence for a role of preganglionic sympathetic neurons (all of which are cholinergic; Figure 1) in modulating immune responses is nearly all indirect. One exception is our recent demonstration of the strong anti-inflammatory action mediated by the preganglionic sympathetic neurons of the splanchnic nerves in endotoxemic rats [28]. Another is the demonstration that the anti-inflammatory action of bee venom is mediated by preganglionic neurons to the adrenal medulla in mice [12]. By contrast, the evidence for a strong immunomodulatory role of sympathetic postganglionic neurons (most of which are noradrenergic) is overwhelming. This has been well reviewed elsewhere [30,32].

In the context of arthritis, several technical factors prevent us directly inferring the roles of preganglionic sympathetic nerves from those of postganglionic sympathetic nerves. First, not all the actions of postganglionic sympathetic nerves depend on preganglionic neural inputs. Janig and Green [58] have recently reviewed their studies on bradykinin-evoked plasma extravasation in the rat knee joint, which clearly showed that this inflammatory response depended in large measure on the presence of sympathetic nerve terminals in the joint but not on their neural activity or their preganglionic inputs. They postulated that this was due to ongoing, non-synaptic release from the terminals of inflammatory mediators such as prostaglandins [58]. Second, the standard approach used to investigate the immunomodulatory role of sympathetic nerves is to destroy their terminals with the toxin, 6-hydroxydopamine (6-OHDA). Unfortunately this also destroys catecholaminergic immune cells, which play an increasingly dominant role over the time course of arthritis as local noradrenergic terminals withdraw [59]; therefore, interpretation of its effects is complicated. Third, chemical sympathectomy with systemic 6-OHDA in the presymptomatic phase of arthritis lessens disease severity but this treatment during the established phase worsens the disease [60], suggesting a bimodal action. Fourth, local and systemic sympathetic nerves may have opposing actions. In rats given adjuvant-induced arthritis, Lorton and colleagues [61,62] injected 6-OHDA into the lymph nodes that drain the hindlimbs, which sympathectomised internal organs, including the spleen, but preserved the sympathetic innervation in the limbs. This worsened hindlimb arthritis, even when given presymptomatically. By contrast, systemic 6-OHDA sympathectomised the affected limbs and reduced disease severity.

In summary, noradrenergic postganglionic sympathetic nerves in the joint may have pro-inflammatory actions but it is unclear whether any of this is attributable to the actions of (cholinergic) preganglionic sympathetic nerves (Figure 1). The data suggest that those pro-inflammatory actions are local to the affected joints. A systemic anti-inflammatory action of sympathetic nerves to internal organs such as the spleen is more likely to be driven by preganglionic sympathetic neurons [28,30], though this has yet to be tested in the context of arthritis.

## Non-neural cholinergic pathways, endotoxemia and macrophages

Among the proinflammatory cytokines, TNF appears to play a pivotal role in lethal endotoxemia [63]. As indicated above, regulation of its levels in rodents by vagus nerve stimulation or dissection in experimental endotoxemia was pivotal early evidence linking the cholinergic pathway to inflammation [6]. Furthermore, nicotine or choline suppressed systemic splenic TNF production in endotoxemic mice, the effect of choline being abolished in α7nAChR knock-out mice [14,64].

Since monocytes/macrophages would appear to be a major source of TNF in response to endotoxin, a number of *in vitro* studies have been carried out to test the effects of cholinergic agonists on cytokine production in stimulated monocytes/macrophages. It has been reported that in stimulated human monocyte-derived macrophages acetylcholine, choline, nicotine and other agonists inhibited pro-inflammatory cytokine release through an α7nAChR-dependent mechanism [6,64-68] - these data for TNF formation are compiled in Table 1. Similar TNF data were found in human monocytes [69,70], mononuclear cells [65] and whole blood [64,71], although the nicotine data could not be confirmed for the monocytes and whole blood [72] (Table 1).

**Table 1:** **Effect of cholinergic agonists on tumour necrosis factor formation in stimulated monocytes/macrophages**

**Cell type**	**Agonist**	**TNF levels**	**Reference**
**Human**			
Monocyte-derived macrophages	Acetylcholine, nicotine	↓	[6]
	Choline	↓	[64]
	Nicotine	↓	[66]
Monocytes	Nicotine	↓	[70]
	Nicotine, cotinine	↓	[69]
	Nicotine	X	[72]
	GTS-21	↓	
	Nicotine	↓	[65]
(mononuclear cells) Whole blood	Choline	↓	[64]
	Nicotine	X	[72]
	GTS-21	↓	
(rheumatoid arthritis)	Nicotine, GTS-21	↓	[71]
**Murine**			
Resident peritoneal macrophages	Nicotine	↓	[13]
	Nicotine, AR-R17779	↓	[74]
	Nicotine	↓	[73]
	GTS-21	↓	[84]
Elicited peritoneal macrophages	Nicotine, acetylcholine	↓	[17]
	Choline	↓	[64]
	Acetylcholine	↓	[75]
	Nicotine	↓	[76]

X = No change. TNF, tumour necrosis factor.

Both stimulated resident and elicited murine peritoneal macrophages also gave positive findings for suppression of cytokine secretion by the same cholinergic agonists [13,17,73-76] via a proposed Jak2/STAT3 mechanism [13]. However, the more specific α7nAChR agonist, AR-R17779, was strangely less potent than nicotine, implying that nicotinic inhibition of macrophage activation may involve other receptors in addition to α7nAChR [74,75]; this is consistent with findings that macrophages express several subtypes of nAChR [77]. These data for TNF formation by murine macrophages are also listed in Table 1. There are obviously some literature data that need to be reconciled.

## Non-neural cholinergic signalling in arthritis

Inflammatory cytokine production in RA whole blood cultures was suppressed by cholinergic agonists [71]. α7nAChR is expressed in the RA synovium, mainly by intimal lining synoviocytes [78]; it is also found in fibroblast-like synoviocytes *in vitro* [78] and its stimulation in these cells led to potent inhibition of proinflammatory cytokine formation [79]. RA and osteoarthritis synovial biopsies had choline acetyltransferase expression in both the fibroblast-like synoviocytes and mononuclear cells, and it has been suggested that local acetylcholine production (Figure 1) could be contributing to the regulation of joint inflammation by the ‘cholinergic anti-inflammatory pathway’ [80].

**Figure 2:**
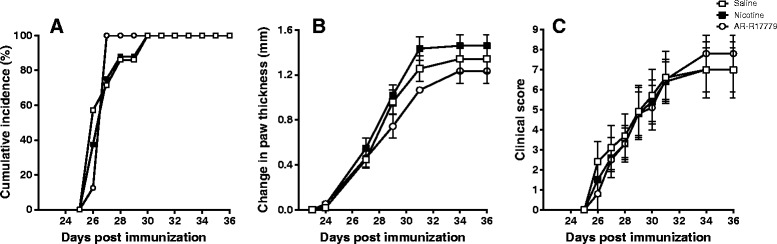
**Nicotine and AR-R17779 fail to ameliorate collagen-induced arthritis (CIA).** Male DBA/1 mice (6 to 8 weeks) were immunized for CIA on day 0 (100 μg chick type II collagen in complete Freund’s adjuvant containing 5 mg/ml of heat-killed *Mycobacterium tuberculosis*), followed by a booster injection on day 21 [85]. Beginning on day 21, mice were treated intraperitoneally with nicotine (400 μg/kg), AR-R17779 (5 mg/kg) or vehicle (saline), twice daily for 7 days. **(A)** Cumulative incidence (percentage). **(B)** Change in paw thickness (calliper). **(C)** Clinical score (0 to 4 per paw; maximum score of 16 per mouse). Data are expressed as mean ± standard error of the mean; n = 8 mice per group.

As noted above, it has been reported that the less specific α7nAChR agonist, nicotine, and the more specific agonist, AR-R17779, ameliorated and/or delayed murine collagen-induced arthritis [48,49]. In addition, this arthritis in α7nAChR−/− mice was more severe and associated with increased proinflammatory cytokine formation [50]. However, contradictory data in these knock-out mice have been presented in the same model [81]. Nicotine pre-treatment aggravated adjuvant arthritis in rats whereas post-treatment suppressed the disease [82]. In our hands, however, both nicotine and AR-R17779, at concentrations similar to those in [48], failed to suppress murine collagen-induced arthritis (Figure 2). Such divergent observations again remain to be reconciled. What might help is a thorough analysis of the expression of the different nicotine-binding receptors in various arthritis models.

## Conclusion

It is clear that centrally acting muscarinic agonists, electrical stimulation of the vagus to activate preganglionic parasympathetic nerves, and treatment with nAChR agonists can all act systemically (though not necessarily identically) to reduce the production of inflammatory cytokines presumably mostly by macrophages. The full pathways by which they work are complex and incompletely understood. Systemic treatment with nicotinic agonists has been reported to reduce the incidence and severity of murine arthritis, although we did not confirm this finding. Sympathetic preganglionic neurons also have systemic anti-inflammatory actions that suppress the production of inflammatory cytokines. A key difference is that the sympathetic pathway is activated reflexively by peripheral inflammation while the vagal pathway appears not to be. The actions of sympathetic preganglionic neurons on arthritis may be more complex, however, and need to be clarified.

It is important that further studies are carried out in this area since clinical trials based upon the concept of the ‘cholinergic anti-inflammatory pathway’ have been proposed and/or are underway [83]. At present, targeting sympathetic or adrenergic processes with drugs is less attractive because their actions may be helpful or unhelpful, depending on the disease stage [60]. Also, the conflict between the actions of local versus systemic sympathetic nerves needs to be untangled for future progress along this line.

Much less convincing in our view is the idea that deficient control by the ‘cholinergic ant-inflammatory pathway’ is a significant factor leading to the onset or exacerbation of arthritis [47]. The evidence so far indicates that there is no ongoing tone in the vagal ‘cholinergic anti-inflammatory pathway’ and that it is not the efferent arm of the inflammatory reflex: sympathetic pathways play that role [28,30]. The loss of cardiac vagal tone in RA and other inflammatory conditions is a predictable consequence of peripheral inflammation rather than a cause. Until there is convincing evidence to the contrary, we suggest that it could mislead us if we uncritically embrace the ‘cholinergic anti-inflammatory pathway’ as a significant factor in the pathogenesis of RA.

## Note

This article is part of the series ‘*At the interface between immunology and neurology in rheumatic diseases’*, edited by Rainer Straub. Other articles in this series can be found at http://arthritis-research.com/series/neurology

## Abbreviations

6-OHDA: 6-hydroxydopamine
HF-HRV: High frequency heart rate variability
LPS: Lipopolysaccharide
nAChR: nicotinic acetylcholine receptor
RA: Rheumatoid arthritis
TNF: Tumour necrosis factor
